# The Oklahoma Falls Prevention Program Targeting Rural Community-Dwelling Older Adults

**DOI:** 10.1093/geroni/igaa057.967

**Published:** 2020-12-16

**Authors:** Keith Kleszynski, Janis Campbell, Omolara Henley, Lee Jennings

**Affiliations:** 1 University of Oklahoma Health Sciences Center, Oklahoma City, Oklahoma, United States; 2 University of Oklahoma, Oklahoma City, Oklahoma, United States; 3 University of Oklahoma Health Science Center/OHAI, Oklahoma City, Oklahoma, United States

## Abstract

The Oklahoma Healthy Aging Initiative is a statewide health promotion program for older adults based at the University of Oklahoma. Seven staff educators and 32 volunteers delivered 2 community-based fall prevention programs, Staying Active and Independent for Life (SAIL) and Tai Chi Quan: Moving for Better Balance (TCBB) to 763 older Oklahomans in 71 sites across the state over 9 months. For both programs, twenty-four 60-90 minute classes were delivered over 12 weeks with pre and post assessments completed at the first and last class, respectively. Two hundred ninety eight participants (39%) completed at least 75% of class sessions and to date 140 completed a posttest evaluation and were included in the evaluation. Participants were mostly older (87% ≥60 years), female (86%), college educated (45%), white (87%), and most participated in TCBB (89%). Participants improved in 2 physical performance measures: mean 30-second chair stands increased from 11.5 (SD3.8) to 13.1 (SD3.4) stands (p<0.0001); and mean timed up and go time decreased from 10.0 (SD2.9) to 9.4 (SD2.9) seconds (p=0.004 ). More participants reported vigorous or moderate activity at least 3 times per week after program completion, 134 (96%) vs. 114 (81%), p=0.0001. There was no difference in measures of global health, satisfaction with social roles and activities, or companionship with participant mean scores near the upper range of these scales at baseline. Older Oklahomans participating in community-based exercise report good overall health and report high social connection. Future efforts will focus on more socially isolated older adults and diverse communities.

